# Comparing mitral transcatheter edge-to-edge repair and surgical intervention in mitral regurgitation: A meta-analysis

**DOI:** 10.34172/jcvtr.026.33487

**Published:** 2026-03-30

**Authors:** Emídio Mata, Barbara Lage Garcia, Margarida Castro, Mariana Tinoco, Luísa Pinheiro, João Português, Francisco Ferreira, Lucy Calvo, Sílvia Ribeiro, António Lourenço

**Affiliations:** Cardiology Department, Unidade Local de Saúde de Alto Ave, Guimarães, Portugal

**Keywords:** Mitral valve insufficiency, Mitral regurgitation, Mitral transcatheter edge-to-edge repair, Percutaneous mitral valve repair, Surgical mitral valve repair, Mitral valve replacement

## Abstract

Surgery remains the standard treatment for severe mitral valve regurgitation (MR), but growing evidence highlights the potential role of mitral valve percutaneous edge-to-edge repair (M-TEER). This meta-analysis aims to compare 12-month all-cause mortality between M-TEER and surgical intervention (SMVI). A systematic search (October 2024) of PubMed, Cochrane, Scopus, and Web of Science identified randomized control trials (RCTs) and propensity-matched observational studies comparing 12-month all-cause mortality in MR patients treated with M-TEER or SMVI. An inverse variance random-effects meta-analysis assessed outcomes using risk ratios (RR) and 95% confidence intervals (CI). Two RCTs (MATTERHORN and EVEREST II) and three observational studies totaling 1,782 patients, were included in the final analysis. A non-significant trend of higher mortality at 30 days was observed in the SMVI group (RR: 0.72; CI: 0.26–2.00), along with higher complication rates mainly due to bleeding. At 12 months follow-up, SMVI was associated with a significantly lower risk of all-cause mortality (RR: 1.41; CI: 1.11–1.81), while the M-TEER group had more patients with MR grade 3 or higher (RR: 4.05; CI: 1.54–10.67), with a non-significant trend of higher rate of MR reintervention (RR 2.51; CI 0.83–7.66) at 12 months. Based on current evidence, M-TEER should continue to be reserved for patients with prohibitive high surgical risk. While propensity-matched cohorts were pooled for the study estimates, the limited available data from randomized trials, combined with heterogeneity in patient populations, particularly regarding MR etiology, underscores the need for further studies.

## Introduction

 Mitral valve regurgitation (MR) is among the most common valve disorders, affecting approximately 2% of the global population worldwide.^1^ It occurs equally in males and females and increases in prevalence with age. MR is classified as either primary MR (PMR), caused by structural abnormalities in the leaflets, chordae or papillary muscles; or as secondary MR (SMR), resulting from left ventricular (LV) wall motion abnormalities or remodeling, without intrinsic valve defects.^1^

 Patients with PMR usually have preserved left ventricular ejection fraction (LVEF) and generally lower surgical risks, though some may have reduced LVEF, and thus face worst surgical outcomes.^2^ In contrast, SMR is often associated with heart failure (HF) in about 30% of patients, with a higher surgical risk.^3^

 While surgical intervention is the standard approach for severe MR, advancements have led to the development of mitral valve transcatheter edge-to-edge repair (M-TEER) as a less invasive option for high-risk patients.

 The EVEREST II trial marked a landmark as the first randomized controlled trial (RCT) to compare long-term mortality M-TEER with surgical mitral valve intervention (SMVI) in patients with clinically significant mitral regurgitation who were eligible for surgery.^4^ The study revealed that M-TEER offered a superior safety profile while showing no significant differences in 12-month mortality when compared to SMVI.

 Recently, a new RCT, the MATTERHORN trial,^5^ provided new insights, comparing M-TEER and surgical mitral valve repair in patients with SMR, concluding that M-TEER was not significantly different to SMVI regarding major adverse events at 12 months.

 This systematic review with meta-analysis aims to compare mortality and immediate safety outcomes of patients with significant MR undergoing either M-TEER or SMVI by pooling results from these RCTs while also incorporating data from propensity score-matched studies to enrich the pooled population.

## Methods

###  Search Strategy and Selection Criteria

 This study was designed following Preferred Reporting Items for Systematic Reviews and Meta-Analyses (PRISMA) statement,^6^ and registered on PROSPERO (CRD42024611622).

 On October 11, 2024, a systematic search from inception PubMed, Cochrane Central Register of Controlled Trials, Scopus and Web of Science was conducted. The search encompassed broad terms referring to “transcatheter mitral valve repair”, “mitral valve surgery” and “mitral regurgitation” (Full query in Supplementary Material Table 1). The references’ lists of the included studies and relevant reviews were searched for additional publications. Eligible studies satisfied the following inclusion criteria: (1) RCT or observational studies with propensity matched cohorts that enrolled (2) patients with significant MR (3) undergoing either M-TEER or SMVI (4) and comparing all-cause mortality at 12 months of both groups, expressed as odds ratio, freedom from outcome or risk ratio. Single arm studies were excluded. No restrictions were applied for publication status or publication language.

###  Selection Process

 Two reviewers independently screened all titles and abstracts for potential eligibility after duplicate records were removed. To ensure sensitivity at the abstract-level selection, full texts were retrieved in all cases of unclear abstracts or disagreement between reviewers. Full texts of potentially eligible studies were then retrieved and assessed independently by the same two reviewers to determine inclusion based on the predefined criteria described above. Discrepancies at the full-text level were resolved through discussion with a third reviewer. No automation tools were used in any phase of the screening process. All references were managed using EndNote X9 (Clarivate Analytics).

###  Data Extraction and Outcomes of Interest

 Two reviewers independently extracted data from each eligible study, using a standardized data extraction form with information regarding study and patient characteristics, MR etiology, medical or device therapy at baseline, pre-procedure echocardiographic features, and outcomes at 30 days and 12 months.

 All-cause mortality at 12 months was the main outcome of interest. Secondary outcomes were all-cause mortality at 30 days, short-term complications, New York Heart Association (NYHA) functional classification, MR grade and mitral valve (MV) reintervention at 12 months.

###  Risk of Bias

 The risk of bias was conducted using the Cochrane risk of bias tool for RCTs and for Non-randomized Studies - Interventions, Version 2 (ROBINS-I V2 TOOL). The presence of publication bias could not be performed because only five studies were included.

###  Synthesis Methods

 A narrative synthesis approach was primarily employed to describe and interpret findings across studies, particularly when analysis was limited due to heterogeneity in study designs, outcome reporting, populations, or outcome definitions. Structured tables grouped by intervention (M-TEER vs. SMVI) were used to present baseline characteristics, clinical outcomes, and risk of bias assessments in a standardized format to facilitate meaningful comparison across studies.

 Where outcomes were sufficiently homogeneous, a study-level meta-analysis was conducted following an intention-to-treat approach. Outcomes such as all-cause mortality and MV reintervention were treated as cumulative events and analyzed using event rates at defined time points. In contrast, outcomes such as NYHA functional class and MR grade were assessed using cross-sectional prevalence at specific time points, with the number of patients still under follow-up at that time used as the denominator. For observational studies that reported only event proportions without time-to-event data, the baseline cohort size was used as the denominator unless censoring or loss to follow-up was explicitly addressed. All estimates were calculated using a random-effects model based on the DerSimonian and Laird method via Review Manager version 5.4, The Cochrane Collaboration. The effect measure was expressed as risk ratios (RR) with 95% confidence intervals (CI), analyzed using the generic inverse variance method. Heterogeneity was tested and quantified using Chi-squared test and I^2^ statistics. Thresholds of I^2^ statistic of 25% (low), 50% (moderate) and 75% (high) were defined. The statistical level of significance was two-tailed *P*-value < 0.05.

## Results

###  Search Results

**Figure 1:**
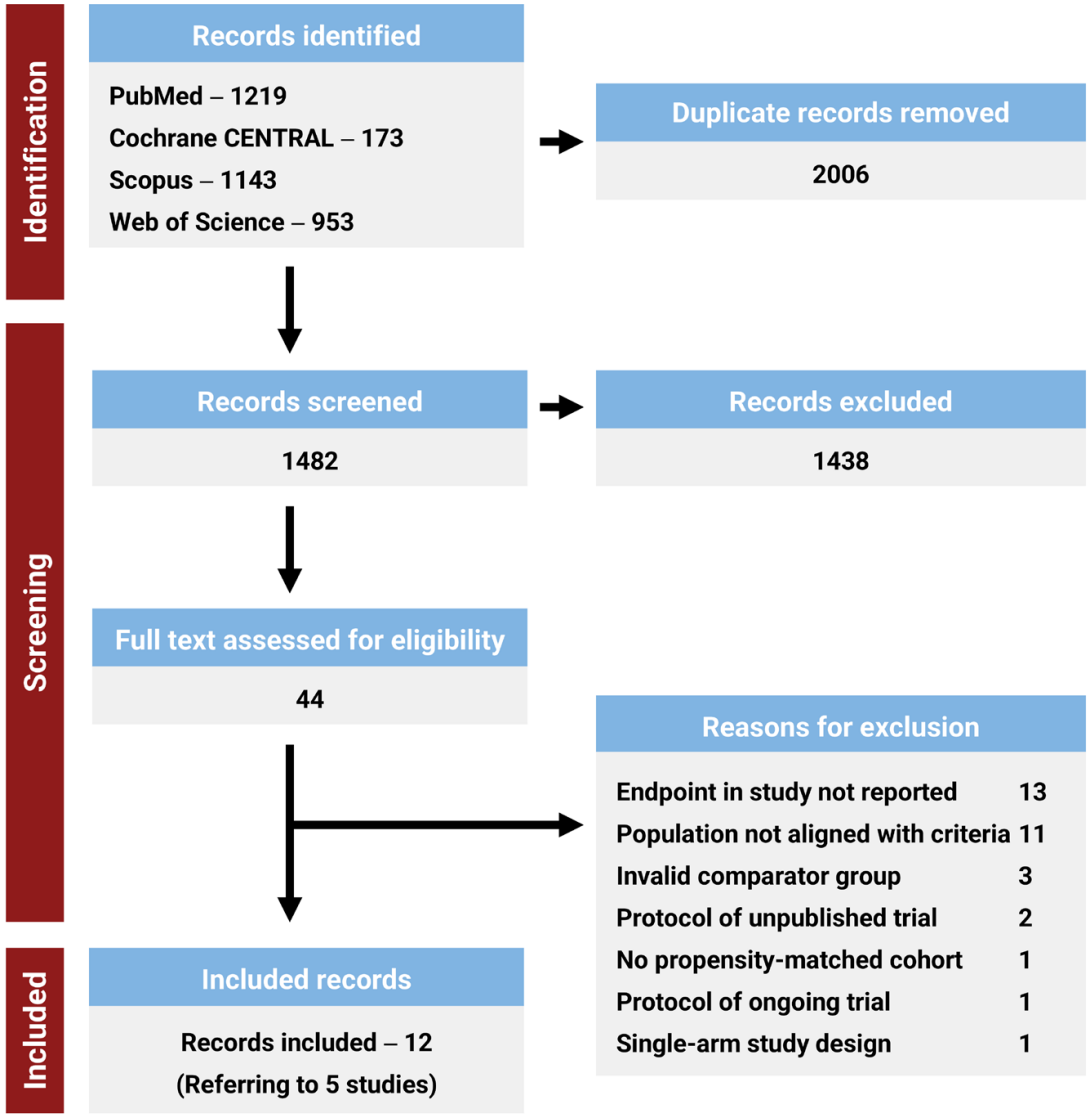
PRISMA Flowchart.Study identification and selection process through the different stages of the systematic review. Cochrane CENTRAL: Cochrane Central Register of Controlled Trials

 The literature search (Figure 1) identified 1482 articles after duplicate removal. Of these, 12 records regarding a total of five studies were included for final analysis. Of the five studies included, two were RCTs: the recently published MATTERHORN trial, ^5^ and the EVEREST II trial,^4^ represented by eight records with three publications reporting outcomes at different follow-ups.^4,7,8^ The remaining three were observational studies.^9-11^

###  Characteristics of Included Studies

 Characteristics of the included studies are shown in Table 1. They were published between 2011 and 2024, across North America and Europe.

**Table 1:** Included studies characteristics

**Study,** **Study type,** **Country,** **No. of centers, Recruitment Period**	**Inclusion/Exclusion criteria, ** **Propensity match variables**	**Number of patients**		**Mitral valve regurgitation etiology (%)**				**Surgical repair (%)**
				**M-TEER group**		**Surgery group**		
		**M-TEER**	**Surgery**	**PMR**	**SMR**	**PMR**	**SMR**	
**EVEREST II,** 2011 RCT Canada, USA 37 centers Sep 2005 to Nov 2008	**Inclusion criteria:** − Moderate to severe PMR or SMR (EROA ≥ 0.3 cm2; regurgitant volume ≥ 45 ml; regurgitation fraction ≥ 40%; *vena contracta* ≥ 0.5cm) − Eligible for both M-TEER and surgery − Symptomatic: LVEF > 25% or LVESDD ≤ 55 mm − Asymptomatic: LVEF 25-60% or LVESDD ≥ 40 mm, or new-onset AF, or PHT **Exclusions criteria:** − Need for other cardiac surgery − Myocardial infarction in the prior 12 weeks; endocarditis; rheumatic heart disease; serum creatinine > 2.5 mg/dl − Mitral valve mitral valve area < 4 cm2, leaflet flail width ≥ 15 mm and gap ≥ 10 mm, leaflet tethering/coaptation depth > 11 mm and length < 2 mm.	184	95	135/184 (73.4)	49/184 (26.6)	69/95 (72.6)	26/95 (27.4)	69/80 (86.3)
**MATTERHORN,** 2024 RCT Germany 16 centers Feb 2015 to Dec 2022	**Inclusion criteria:** − Significant SMR (At least 2 of: EROA ≥ 20 mm^2^; regurgitant volume ≥ 30 ml; regurgitant fraction ≥ 50%; *vena contracta* width > 8 mm) or 2 HHF in the 12 months prior − Eligible for both M-TEER and surgery − LVEF ≥ 20% and NYHA class II to class IV **Exclusion criteria:** − Additional severe valvular disease − Coronary revascularization or CRT therapy within 1 month prior	104	104	0/104 (0.0)	104/104 (100)	0/104 (0.0)	104/104 (100)	72/100 (72.0)
**Koschutnik,** 2022 Observational Austria 1 center Jul 2017 to Apr 2020	**Inclusion criteria:** − Moderate to severe PMR or SMR **Propensity match variables: **age; EuroSCORE-II; LVEF; serum NT-proBNP levels	51	51	20/51 (39.2)	31/51 (60.8)	43/51 (84.3)	8/51 (15.7)	34/51 (66.7)
**Amabile,** 2023 Observational USA 58 centers Jan 2015 to Dec 2020	**Inclusion criteria:** − Significant ischemic MR requiring intervention **Exclusion criteria:** − Rheumatic heart disease; non-ischemic MR etiology; periprocedural period with acute myocardial infarction or papillary muscle rupture − Patients who underwent MV replacement via surgery or through catheter **Propensity match variables:** 29 variables (demographics; comorbidities; surgical history; use of CV, anti-platelet and anticoagulant medications; b-type natriuretic peptide; LVEF)	550	550	0/550 (0.0)	550/550 (100)	0/550 (0.0)	550/550 (100)	550/550 (100.0)
**Silaschi,** 2024 Observational Germany 1 center Jan 2010 to Dec 2021	**Inclusion criteria:** − Moderate to severe PMR or SMR requiring intervention **Exclusion criteria:** − Surgical procedures via median sternotomy; patients with endocarditis **Propensity match variables: **age; EuroSCORE-II; LVEF	49	49	40/49 (81.6)	9/49 (18.4)	40/49 (81.6)	9/49 (18.4)	48/49 (98.0)*

AF: atrial fibrillation; CRT: cardiac resynchronization therapy; CV: cardiovascular; EROA: effective regurgitant orifice area; HHF: hospitalization related to heart failure; LVEF: left ventricle ejection fraction; LVESDD: left ventricle end-systolic diameter dimension; MR: mitral valve regurgitation; M-TEER: Mitral valve transcatheter edge-to-edge repair; NYHA: New York Heart Association; PHT: pulmonary hypertension; PMR: primary mitral regurgitation; RCT: randomized controlled trial; SMR: secondary mitral regurgitation. * Minimally invasive surgery repair.

 EVEREST II,^4^ conducted in 37 North American centers (2005–2008), enrolled patients with moderate-to-severe PMR or SMR eligible for both procedures, excluding those with recent myocardial infarction, endocarditis, or unfavorable valve anatomy. MATTERHORN,^5^ conducted in 16 German centers (2015–2022), included symptomatic patients with significant SMR, excluding additional severe valvular disease, recent revascularization or LVEF less than 20%.

 Among the observational studies, two were prospectively recruited and retrospectively analyzed,^9,10^ while one was purely retrospective.^11^ Koschutnik and Silaschi focused on patients with PMR or SMR using matched cohorts based on age, EuroSCORE-II, and LVEF, while Koschutnik also including NT-proBNP levels. Amabile, utilizing the TriNetX database across 58 U.S. centers, employed International Classification of Diseases, Tenth Revision (ICD-10) and Current Procedural Terminology (CPT) codes to identify patients with SMR by selecting those with MR and ischemic heart disease.

 The five studies collectively recruited 1787 patients (347 with PMR and 1440 with SMR), of whom 938 underwent M-TEER and 849 underwent SMVI (82.1% receiving MV repair). All studies focused on MV repair, with one excluding patients who underwent MV replacement,^11^ and another exclusively including minimally invasive surgery.^9^ Details of the percutaneous and surgical techniques and devices are provided in Supplementary Material Table 2.

 The quality assessment, summarized in Supplementary Material Tables 3 and 4, indicated that the studies were of overall good quality.

###  Baseline Characteristics of the Patients

 The baseline characteristics of the study population are summarized in Table 2. Male patients predominated in both the M-TEER and SMVI groups (63.2% and 61.7%, respectively), with a comparable mean age (71.3 ± 10.6 vs. 71.1 ± 9.6 years). Patients undergoing M-TEER had a slightly higher EuroScore-II risk profile (3.83 ± 3.79% vs. 3.23 ± 2.72%).

**Table 2:** Study population characteristics

	**Age, years (n) **		**Male (%)**		**EuroSCORE II, % (n) **		**Previous cardiac surgery (%)**		**NYHA class≥III (%)**		**MR grade≥3 (%)**		**EROA, mm** ^2^ ** (n) **		**LVEF, % (n) **		**LVEDV, mL (n) **	
	**M-TEER**	**Surgery**	**M-TEER**	**Surgery**	**M-TEER**	**Surgery**	**M-TEER**	**Surgery**	**M-TEER**	**Surgery**	**M-TEER**	**Surgery**	**M-TEER**	**Surgery**	**M-TEER**	**Surgery**	**M-TEER**	**Surgery**
**EVEREST II,** 2011	67.3 ± 12.8 (184)	65.7 ± 12.9 (95)	115/184 (62.5)	63/95 (66.3)	-	-	38/184 (20.7)*	18/95 (18.9)*	94/184 (51.1)	45/95 (47.4)	176/184 (95.7%)	88/95 (92.6%)	0.56 ± 0.38 (171)	0.59 ± 0.35 (87)	60.0 ± 10.1 (182)	60.6 ± 11.0 (95)	159.03 ± 37.33 (144)	160.39 ± 46.66 (66)
**MATTERHORN,** 2024	70.2 ± 8.1 (102)	70.9 ± 7.8 (101)	66/104 (63.5)	59/104 (56.7)	3.0 [1.6; 4.5] (96)	3.0 [1.8; 4.2] (92)	4/103 (3.9)*	3/103 (2.9)*	84/102 (82.4)	90/101 (89.1)	99/102 (97.1%)	92/97 (94.8%)	0.2 ± 0.1 (86)	0.2 ± 0.1 (76)	42.6 ± 10.9 (100)	43.4 ± 12.5 (93)	170.0 ± 58.2 (100)	158.8 ± 56.6 (93)
**Koschutnik,** 2022	74.0 ± 9.0 (51)	71.1 ± 10.1 (51)	18/51 (35.3)	22/51 (43.1)	6.8 ± 5.7 (51)	5.0 ± 3.9 (51)	9/51 (17.6)*	0/51 (0.0)*	44/51 (86.3)	39/51 (76.5)	-	-	-	-	52.3 ± 16.1 (51)	57.9 ± 13.8 (51)	-	-
**Amabile,** 2023	72.6 ± 10.1 (550)	72.1 ± 9.1 (550)	366/550 (66.6)	356/550 (64.7)	-	-	365/550 (66.4)	362/550 (65.8)	-	-	-	-	-	-	44.2 ± 16.5 (550)	42.3 ± 17.5 (550)	-	-
**Silaschi,** 2024	71.7 ± 8.35 (49)	70.0 ± 7.31 (49)	28/49 (57.1)	24/49 (49.0)	2.3 ± 1.39 (49)	1.8 ± 1.39 (49)	4/49 (8.2)	0/49 (0.0)	15/19 (78.9)	21/43 (48.8)	22/24 (91.7%)	19/23 (82.6%)	0.38 ± 0.21 (49)	0.42 ± 0.24 (49)	60.0 ± 8.0 (49)	60.5 ± 9.7 (49)	113.3 ± 44.9 (49)	125.8 ± 54.0 (49)
**Pooled Total**	71.3 ± 10.6 (936)	71.1 ± 9.6 (846)	593/938 (63.2)	524/849 (61.7)	3.83 ± 3.79 (196)∫	3.23 ± 2.72 (192)∫	420/937 (44.8)	383/848 (45.2)	237/356 (66.6)	195/290 (67.2)	297/310 (95.8)	199/215 (92.6)	0.43 ± 0.34 (306)	0.41 ± 0.31 (212)	48.4 ± 16.1 (932)	46.5 ± 17.4 (838)	155.1 ± 50.4 (293)	151.5 ± 54.7 (208)

Values are expressed as mean ± standard deviation or median and [interquartile range]. CABG: coronary­ artery bypass graft; EROA: effective regurgitant orifice area; LVEDV: left ventricle end-diastolic volume; LVEF: left ventricle ejection fraction; MR: mitral valve regurgitation; M-TEER: mitral valve transcatheter edge-to-edge repair; NYHA: New York Heart Association. * Previous CABG only. ∫ Pooled total using estimated mean and standard deviation from median and interquartile range of MATTERHORN trial.

 Significant symptomatic heart failure (NYHA class III or higher) at baseline was similarly prevalent in both groups (66.6% vs. 67.2%). Most patients had at least MR grade 3, though this was more frequent in the M-TEER group (95.8% vs. 92.6%), with slightly larger effective regurgitant orifice area (EROA) values. Mean LVEF was nearly identical (48.4 ± 16.1% vs. 46.5 ± 17.4%), while left ventricular end-diastolic volume (LVEDV) was marginally higher in the M-TEER group (155.1 ± 50.4 vs. 151.5 ± 54.7 mL).

 Comorbidities were similar across groups (Supplementary Material Table 5), though coronary artery disease was more common in M-TEER patients (43.0% vs. 35.9%). Patients treated with M-TEER also demonstrated higher usage of disease-modifying therapies (Supplementary Material Table 6).

###  Short-Term at 30 Days Outcomes and Complications

 The surgical group experienced a higher incidence of complications at 30 days compared to the M-TEER group (Table 3). This was primarily driven by a significantly greater occurrence of major bleeding (8.9% vs. 30.9%), strokes (0.6% vs. 3.0%), and new-onset atrial fibrillation (AF) (1.8% vs. 13.6%). Patients in the SMVI group also exhibited higher rates of organ failure, including prolonged mechanical ventilation (over 48 hours), deep wound infections, and renal failure requiring dialysis. Notably, the reintervention rates at 30 days were similar between the two groups (5.3% vs. 5.1%).

**Table 3:** Outcomes at discharge or at 30 days

	**All-cause** **mortality (%)**		**Reintervention (%)**		**Major bleeding (%)**		**Stroke (%)**		**New onset AF (%)**		**Myocardial infarction (%)**		**Renal failure (%)**		**Deep wound infection (%)**		**Need of mechanical ventilation>48h (%)**	
	**M-TEER**	**Surgery**	**M-TEER**	**Surgery**	**M-TEER**	**Surgery**	**M-TEER**	**Surgery**	**M-TEER**	**Surgery**	**M-TEER**	**Surgery**	**M-TEER**	**Surgery**	**M-TEER**	**Surgery**	**M-TEER**	**Surgery**
**EVEREST II,** 2011	2/180 (1.1)	2/94 (2.1)	-	1/94 (1.1)	24/180 (13.3)*	42/94 (44.7)*	2/180 (1.1)	2/94 (2.1)	2/180 (1.1)	0/94 (0.0)	0/180 (0.0)	0/94 (0.0)	1/180 (0.6)	0/94 (0.0)	0/180 (0.0)	0/94 (0.0)	0/180 (0.0)	4/94 (4.5)
**MATTERHORN,** 2024	2/101 (2.0)	4/93 (4.3)	5/100 (5.0)	10/91 (11.0)	3/97 (3.1)∫	22/90 (24.4)∫	0/97 (0.0)	4/90 (4.4)	3/97 (3.1)	25/90 (27.8)	0/97 (0.0)	1/91 (1.1)	4/98 (4.1)	9/92 (9.8)	1/100 (1.0)	4/89 (4.5)	4/99 (4.0)	10/92 (10.9)
**Koschutnik,** 2022	-	-	3/51 (5.9)	1/51 (2.0)	-	-	-	-	-	-	-	-	-	-	-	-	-	-
**Amabile,** 2023	-	-	-	-	-	-	-	-	-	-	-	-	-	-	-	-	-	-
**Silaschi,** 2024	3/49 (6.1)	2/49 (4.1)	-	-	2/49 (4.1)†	8/49 (16.3)†	0/49 (0.0)	1/49 (2.0)	-	-	-	-	1/49 (2.0)	4/49 (8.2)	-	-	-	-
**Pooled Total**	7/330 (2.1)	8/236 (3.4)	8/151 (5.3)	12/236 (5.1)	29/326 (8.9)	72/233 (30.9)	2/326 (0.6)	7/233 (3.0)	5/277 (1.8)	25/184 (13.6)	0/277 (0.0)	1/185 (0.5)	6/327 (1.8)	13/235 (5.5)	1/280 (0.4)	4/183 (2.2)	4/279 (1.4)	14/186 (7.5)

AF: atrial fibrillation; M-TEER: mitral valve transcatheter edge-to-edge repair; UGR: use of blood transfusion. * Bleeding requiring 2 or more UGR ∫ Major bleeding according to Valve Academic Research Consortium (VARC) † Bleeding Academic Research Consortium (BARC) 3 or 4

**Figure 2:**
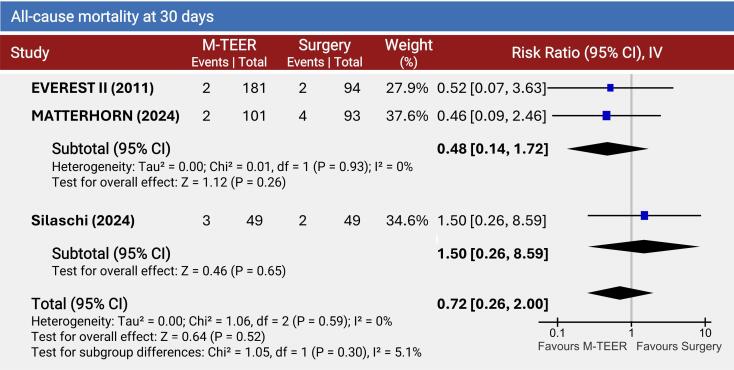
Forest Plot for All-Cause Mortality at 30 Days.This forest plot compares the risk ratio (RR) of all-cause mortality at 30 days between mitral transcatheter edge-to-edge repair (M-TEER) and surgery in three studies: EVEREST II (2011), MATTERHORN (2024), and Silaschi (2024). The combined analysis shows an overall risk ratio of 0.72 (95% CI: 0.26–2.00) favoring M-TEER, though the results are not statistically significant (P = 0.52). *Only includes data from patients with primary mitral regurgitation

 All-cause mortality at 30 days was assessed in three studies, including both RCTs (Figure 2).^4,5,9^ While the data indicated a trend toward higher mortality rates in the SMVI group, particularly in RCTs, no statistically significant differences were observed between the two interventions (RR: 0.72; 95% CI: 0.26–2.00).

###  Twelve-Months Follow-Up Outcomes

**Figure 3:**
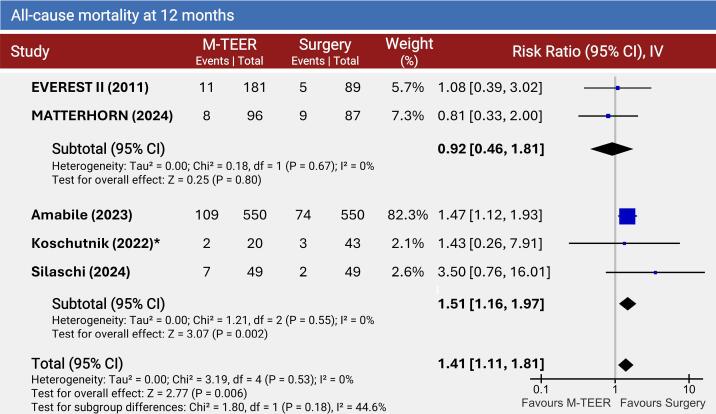
Forest Plot for All-Cause Mortality at 12 months.The forest plot compares all-cause mortality between the intervention group mitral transcatheter edge-to-edge repair (M-TEER) and the surgical mitral intervention (valve repair/replacement) at 12 months across multiple studies. Risk ratios (RR) with 95% confidence intervals (CI) are presented for each study and pooled for two subgroups: randomized control trials and observational studies with propensity matched cohorts. The overall pooled analysis indicates a higher mortality risk in the M-TEER group compared to surgery (RR: 1.41, 95% CI: 1.11–1.81). *Only includes data from patients with primary mitral regurgitation

 At 12 months (Figure 3), patients undergoing M-TEER had a significantly higher risk of all-cause mortality compared to those treated with SMVI (RR: 1.41; 95% CI: 1.11–1.81), a difference largely driven by findings from observational studies. Notably, Amabile demonstrated a significant survival benefit with SMVI at 12 months. Meanwhile, Koschutnik, which included only patients with PMR, favored surgery but without reaching statistical significance. When focusing solely on RCTs, no significant differences were observed at 12 months (RR: 0.92; 95% CI: 0.46–1.81).

**Figure 4:**
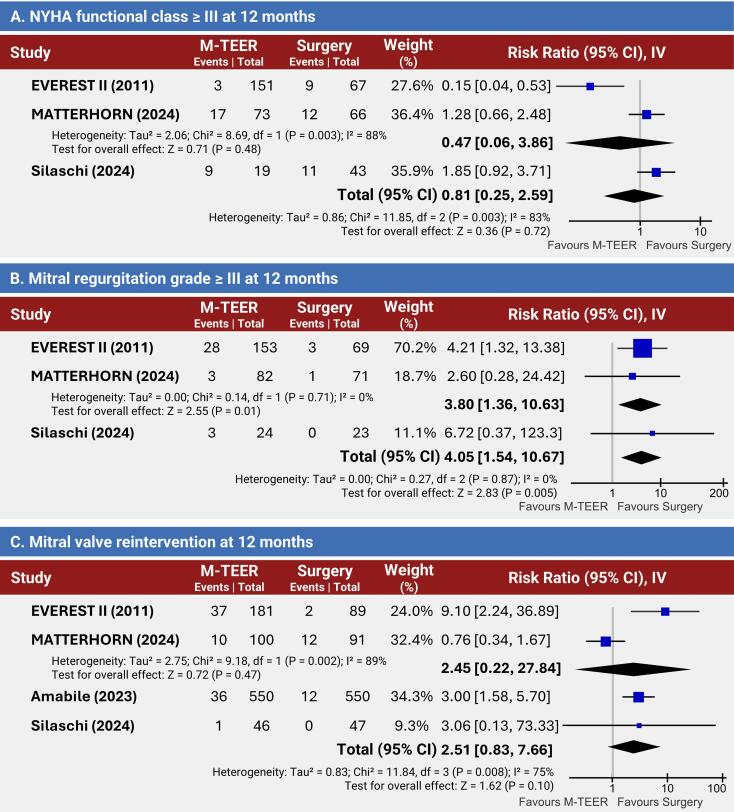
Comparative Outcomes at 12 Months for M-TEER vs. Surgery.(A) Risk ratio (RR) comparing mitral transcatheter edge-to-edge repair (M-TEER) and surgery for the prevalence of NYHA functional class ≥ III at 12 months. Pooled results show no significant difference. (B) RR evaluating the persistence of mitral regurgitation (MR) grade ≥ III at 12 months. The pooled analysis indicates a higher likelihood of residual MR in patients treated with M-TEER compared to surgery. (C) RR for mitral valve reintervention at 12 months across treatment modalities. Results suggest a tendency toward higher reintervention rates in the M-TEER group compared to surgery, although no significant differences were observed in the pooled analysis.

 The proportion of patients who did not achieve NYHA functional class I or II at 12 months was similar between the two groups, as illustrated in Figure 4A. In the EVEREST II trial, patients undergoing SMVI experienced significantly higher rates of symptomatic heart failure. However, this trend was not replicated in the MATTERHORN trial, which reported no significant differences between the groups. Conversely, the Silaschi study found that a greater proportion of patients who underwent surgery remained in NYHA functional class I or II.

 Prevalence of MR grade 3 or higher at 12 months post-procedure was significantly higher in the M-TEER group compared to the SMVI group (RR: 4.05; 95% CI: 1.54–10.67) (Figure 4B). This finding remains unchanged when the observational study is excluded from the analysis (RR: 3.80; 95% CI: 1.36–10.63).^9^

 At 12-month follow-up, MV reintervention rates, depicted in Figure 4C, showed no significant difference between the M-TEER and SMVI groups, although a trend favoring SMVI was noted. In the EVEREST II trial, MV reintervention was significantly more common in the M-TEER group (20.4% vs. 2.2%), a finding also observed in Amabile. In Silaschi, only one patient required reintervention.

## Discussion

**Figure 5:**
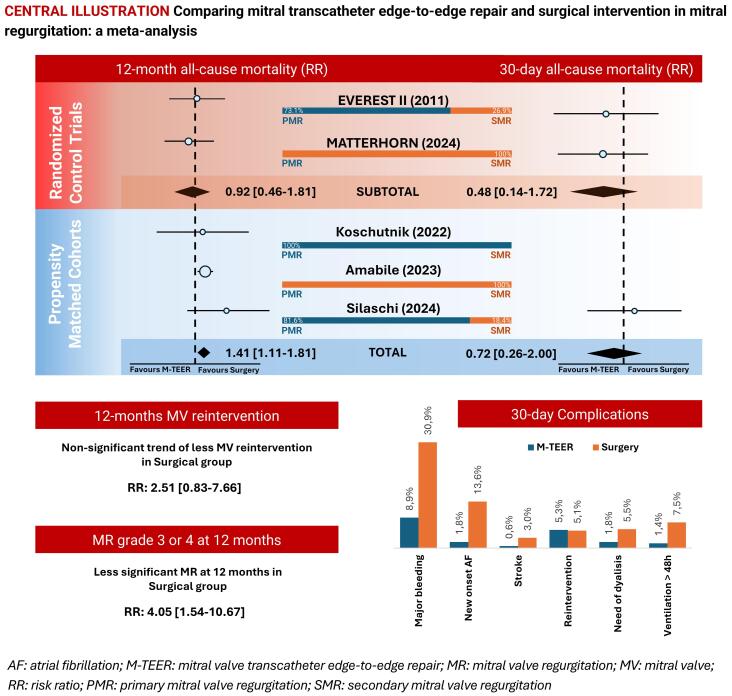
Central Illustration Summarizing the Main Findings

 Over the past decade, M-TEER has emerged as a less invasive approach for patients with prohibitive surgical risk. ^12-14^ According to recent recommendations, M-TEER is now not only considered for patients with SMR and compromised LV function,^15,16^ but has also received a class IIA recommendation for treatment of PMR, as well as SMR, in the newly issued ACC/AHA guidelines.^15^ This upgrade in recommendation for PMR is based on registry data,^17^ as RCTs comparing long-term mortality outcomes between these modalities remain lacking. Given the limited randomized trial data, pooling results from these studies alongside observational studies is necessary to compare interventions effectively (Figure 5). Since M-TEER and SMVI are usually targeted at different patient populations, with M-TEER being reserved for high-risk patients who often have worse prognosis, only propensity-matched cohorts can potentially provide a robust comparison of outcomes, minimizing selection bias from clinical practice.

###  Short-Term Outcomes 

 The findings highlight distinct differences in 30-day outcomes. As expected, surgery is associated with higher early complication rates, while M-TEER demonstrates fewer periprocedural adverse events and comparable reintervention rates, with a nonsignificant trend toward higher mortality in the surgical group.

 The differences in complication rates were primarily driven by a higher incidence of major bleeding, stroke, and new-onset AF in the SMVI group. In fact, transfusions accounted for the largest single component of major adverse events at 30 days and have been previously linked to worse outcomes.^18^ In the EVEREST II trial,^4^ even after excluding transfusion events, the rate of adverse events remained lower in the M-TEER group, seemingly solidifying M-TEER as the better intervention in terms of short-term adverse events.

 Short-term reintervention rates are not significantly different between treatment modalities, but there are differences among trials. The EVEREST II trial did not account for patients with unsuccessful M-TEER procedures who were subsequently referred for surgery in the outpatient setting, thus underestimating M-TEER rates.^4^ However, it is also important to note that during the EVEREST II trial, M-TEER was a novel technique with limited operator experience, resulting in higher failure rates.^4^ The SMVI group may have also underestimated reintervention rates, as other non-mitral valve surgery-related reinterventions in SMVI patients were not accounted for.

 The suggested non-significant trend of higher mortality at 30 days in the SMVI group was primarily driven by RCT data. In contrast, the observational study may favor SMVI due to underling selection bias, even after propensity matching. In the Silaschi cohort,^9^ higher-risk patients may still have been more likely to be in the M-TEER group than in the SMVI group.

###  Twelve-Month Mortality

 Our results indicated that SMVI was favored, with patients who underwent M-TEER exhibiting a significantly higher 12-month mortality risk when compared to surgical treatment. However, no comparable differences were observed when analyzing only the RCTs, with the 12-month mortality risk difference primarily driven by the observational studies.

 As discussed previously, this could be due to underlying selection bias, meaning that the mortality risk may be more closely associated with the patients’ underlying conditions rather than the intervention itself. In a sensitivity analysis, exclusion of the Amabile study, which relied on ICD-10 and CPT codes and was the only purely retrospective study,^11^ resulted in no statistical significance observed (RR 1.18; 95% CI 0.66–2.11) (Supplementary Figure 1). Additionally, five-year mortality rates in the EVEREST II trial showed no significant difference between the groups (20.8% vs. 26.8%; *P* = 0.36), and treatment strategy was not associated with survival in multivariable analysis.^7,8^ It is also important to note that RCTs excluded patients with other severe valve disorders, such as tricuspid regurgitation, which were not excluded in the observational studies and where a considerable prevalence was observed (Supplementary Table 7).

 Age and comorbidity burden naturally contribute to the pooled estimates observed. Although long-term survival in the EVEREST II trial was similar between treatment arms,^4,7,8^ SMR and advanced age were important predictors of decreased survival. While no meta-regression analysis was performed due to the limited data set (5 studies), the apparent impact of SMR prevalence on 12-month mortality suggests that M-TEER may provide better outcomes in patients with SMR compared to those with PMR. Whether the etiology of SMR significantly impacts the mortality effects of M-TEER and SMVI remains unknown.

 These considerations highlight the complexity of interpreting trial and observational data particularly when guiding clinical decisions regarding M-TEER for patients at the threshold of surgical eligibility.

 As for the comparison between M-TEER plus guideline-directed medical therapy (GDMT) and GDMT alone, it has predominantly been studied in SMR. The recently published RESHAPE-HF2 trial,^19^ together with data from the COAPT,^20^ and MITRA-FR trials,^21^ supports the notion that M-TEER reduces heart failure hospitalizations, as confirmed by meta-analysis.^22^ However, its impact on long-term mortality remains inconclusive, with pooled analyses showing only a non-significant trend toward a survival benefit.^22^

###  Functional Class, Mitral Valve Regurgitation Severity and Reintervention at 12 Months

 The proportion of patients not achieving NYHA functional class I or II at 12 months was similar in both groups, though the estimated high heterogeneity due to the varied etiology prevalences across different studies and considerable follow-up loss limit the robustness of these results.

 Prevalence of MR grade 3 or higher at 12 months after intervention was significantly higher in the M-TEER group, even when excluding the observational data.^9^ Although, it should be noted that the M-TEER group had more MR grade 3 or higher with slightly larger EROA values at baseline. The lack of annuloplasty in the percutaneous approach may also partly account for the more severe mitral regurgitation observed in the M-TEER group at 12 months.

 When looking at the MATTERHORN trial,^5^ 98.6% of patients in the surgery group had MR grade 2 or lower at 12 months of follow-up, which was better than anticipated. This difference reflects the trial’s low threshold for MV replacement rather than enforcement of surgical MV repair, which has been associated with higher recurrence rates of significant MR compared to MV replacement in other studies. The technical advances in surgical MV repair strategies and the less-advanced left ventricular disease in that cohort might also help favour surgery.

 MV reintervention at 12-month follow-up showed a non-significant trend toward higher rates in the M-TEER group, favoring surgical treatment. In the EVEREST II trial,^4^ percutaneous treatment was associated with a markedly higher rate of additional procedures for mitral regurgitation (20.4% vs. 2.2%), a finding also observed in Amabile.^11^ Sensitivity analysis excluding the Amabile data demonstrated that the overall effect remained consistent (RR 2.52; 95% CI 0.37–17.15) (Supplementary Figure 2). The EVEREST II trial results may be explained by the limited experience of operators at the time of the trial.^4^ Notably, 28% of M-TEER patients required subsequent MV surgery, particularly within the first 6 months of follow-up. In contrast, re-intervention rates in more recent contemporary studies are much lower, as technical advancements in M-TEER, operator expertise and the development of more effective devices have greatly improved procedural success rates.^23,24^

###  Long-Term Outcomes and Durability of Mitral Valve Interventions

 The long-term outcomes and durability of mitral valve interventions, particularly in the context of the EVEREST II trial, show a nuanced picture. Regarding the 5-year follow-up,^7,8^ despite an initial imbalance in patients with MR grade 3 or higher and a higher rate of surgery for MV dysfunction, few patients experienced worsening MR or required surgery after the 6-month follow-up threshold.

 Despite SMVI demonstrating worse outcomes compared to M-TEER in the first 30 days, SMVI seems to be a better option in terms of reintervention and MR recurrence at 12 months, as most reinterventions resulting from insufficient MR reduction in M-TEER occur within the first 6 months. This trend reflects the nature of clinical failures associated with M-TEER observed in the EVEREST II trial, where most failures occur within the first six months, primarily due to insufficient MR reduction during the initial procedure or early complications. After this period, there was no significant difference in MR occurrence or the need for MV reintervention between the 1-year and 4-year follow-up periods,^7,8^ nor was there differences in mortality or decline in LV systolic function beyond 12 months, despite the higher prevalence of severe residual MR in the M-TEER group at 1 year.^7,8^ These observations linked to the sustained reduction in symptoms and left ventricular dimensions at 5 years,^7,8^ counter concerns that greater residual MR 6 months after successful M-TEER leads to reduced long-term survival. The clinical course after the initial 6-month window provides reassurance that early complications or insufficient MR reduction do not necessarily translate into long-term mortality or functional decline.

 Nevertheless, extended follow-up results from the MATTERHORN trial are anticipated to provide additional data to corroborate these findings, as the EVEREST II trial remains the only study with long-term follow-up to date.

 Several limitations should be acknowledged when interpreting our findings. The clinical trials included in this analysis were conducted at different times, which influenced the experience of operators and the available disease-modifying therapies. Additionally, the varying patient populations and etiologies across these trials limit the external validity of pooled results.

 Observational studies inherently lack randomization, which means certain variables, some of which may not even be identified, are not randomized and can impact the results. While a propensity-matched cohort strategy was employed to minimize the expected heterogeneity of the sample, considerable differences may remain. It is important to note that while surgical risk was used for propensity matching, this risk estimate has not been as reliable for MV repair surgery as it has been for aortic stenosis interventions.^25^ MV repair typically has low operative mortality rates and often uses minimally invasive techniques that contribute to quicker post-operative recovery.^15^

 Significant heterogeneity exists in the patient populations across studies. There are considerable differences in the proportions of MR etiologies among the studies. The M-TEER group had a higher Euro-Score II surgical risk and greater prevalence of coronary artery disease. Additionally, more severe MR was observed in the M-TEER group, along with slightly larger EROA values and marginally higher LVEDV values. The use of different techniques in the comparator groups may further contribute to the heterogeneity of the pooled population, potentially influencing the results and effect estimates. Given this variability, a random-effects model was selected for meta-analytic pooling to account for anticipated differences in treatment effects across studies, rather than assuming a single common effect size as in fixed-effects models. This approach allowed each study to estimate a different, yet related, underlying effect, making it more appropriate in the context of clinical and methodological heterogeneity. It also supported a more conservative and potentially more generalizable interpretation of the findings, particularly when synthesizing evidence from both randomized trials and observational studies. The reliance on study-level summary data further limits the ability to adjust for confounding factors or perform subgroup analyses, thereby restricting cross-trial comparisons.

 Our studies’ endpoint is restricted to 1 year which may be too short to fully appreciate the benefits of surgery when comparing with M-TEER. Extended results from the MATTERHORN trial will certainly add valuable information to the field.

## Conclusion

 In conclusion, a non-significant trend of higher mortality at 30 days was observed in the SMVI group, due to higher complication rates due to bleeding. At 12 months follow-up, SMVI was associated with a significantly lower risk of all-cause mortality, while the M-TEER group had more severe residual MR, with a non-significant trend of higher rate of MR reintervention.

 Based on current evidence, M-TEER should continue to be reserved for patients with prohibitive high surgical risk. While propensity-matched cohorts were pooled for the study estimates, the limited available data from randomized trials, combined with heterogeneity in patient populations, particularly regarding MR etiology, underscores the need for further studies.

## Competing Interests

 No conflicts of interest to declare.

## Ethical Approval

 As this study is a systematic review and meta-analysis, it did not involve direct interaction with human participants or animals, and therefore, no ethical approval or informed consent was required. However, the study adheres to the ethical conduct policy outlined by the International Committee of Medical Journal Editors (ICMJE) (http://www.icmje.org/).

## Supplementary Files

Supplementary file 1 contains Tables S1-S7 and Figures S1-S2.
