# Supplementary material for: Comparing mitral transcatheter edge-to-edge repair and surgical intervention in mitral regurgitation: A meta-analysis
Source: J Cardiovasc Thorac Res. 2026 Mar 30;18(1):4–14. doi: 10.34172/jcvtr.026.33487 (PMC13309324; doi:10.34172/jcvtr.026.33487)
Supplement: Supplementary file 1 — contains Tables S1-S7 and Figures S1-S2. [file jcvtr-18-4-s001.pdf]

J Cardiovasc Thorac Res. 2026;18(1):4-S1
doi:10.34172/jcvtr.026.33487
http://jcvtr.tbzmed.ac.ir

Supplementary Materials

Supplementary Table 1. Full search query

Supplementary Table 2. Device and surgery techniques employed

Supplementary Table 3. Assessment of risk of bias using Cochrane Risk of Bias tool for randomized controlled trials

Supplementary Table 4. Assessment of risk of bias using Cochrane Risk of Bias tool In Non-randomized Studies – Interventions, Version 2

Supplementary Table 5. Study population comorbidities

Supplementary Table 6. Echocardiographic characteristics of study population

Supplementary Table 7. Study population therapeutic management

Supplementary Figure 1. Sensitivity analysis of 12 months all-cause mortality at 12 months

Supplementary Figure 2. Sensitivity analysis of 12 months mitral valve reintervention at 12 months

Supplementary Table 1. Full search query

SEARCH TERMS	
WEB OF SCIENCE	(((TI=("transcatheter") OR TI=("Percutaneous")) AND TI=("Mitral") AND TI=("repair") OR TI=("MitraClip")) AND TI=("mitral regurgitation")AND (((TI=("surgery") OR TI=("surgical")) AND TI=("Mitral") AND TI=("repair")))) OR (((AB=("transcatheter") OR AB=("Percutaneous")) AND AB=("Mitral") AND AB=("repair") OR AB=("MitraClip")) AND AB=("mitral regurgitation")AND (((AB=("surgery") OR AB=("surgical")) AND AB=("Mitral") AND AB=("repair"))))
SCOPUS	(((TITLE-ABS("transcatheter") OR TITLE-ABS("Percutaneous")) AND TITLE-ABS("Mitral") AND TITLE-ABS("repair") OR TITLE-ABS("MitraClip")) AND TITLE-ABS("mitral regurgitation")AND (((TITLE-ABS("surgery") OR TITLE-ABS("surgical")) AND TITLE-ABS("Mitral") AND TITLE-ABS("repair"))))
PUBMED	(("transcatheter"[Title/Abstract] OR "Percutaneous"[Title/Abstract]) AND "Mitral"[Title/Abstract] AND "repair"[Title/Abstract] OR "MitraClip"[Title/Abstract]) AND "mitral regurgitation"[Title/Abstract]AND ((("surgery"[Title/Abstract] OR "surgical"[Title/Abstract]) AND "Mitral"[Title/Abstract] AND "repair"[Title/Abstract]))
COCHRANE CENTRAL	((("transcatheter" OR "percutaneous") AND "mitral" AND "repair" OR "MitraClip") AND "mitral regurgitation" AND ((("surgery" OR "surgical") AND "mitral" AND "repair")))

Supplementary Table 2. Device and surgery techniques employed

	M-TEER Device (%)		Surgery techniques
	MITRACLIP	PASCAL	
**EVEREST II,** 2011	178/178 (100.0)	-	Annuloplasty; Tendinous cord and leaflet repair; Leaflet resection; Valve replacement
**MATTERHORN,** 2024	102/102 (100.0)	-	Annuloplasty; Artificial tendinous cord implantation; Cleft suture; Valve replacement
**Koschutnik,** 2022	46/51 (90.2)	5/51 (9.8)	Annuloplasty and/or chordae tendineae replacement; Valve replacement
**Amabile,** 2023	550/550 (100.0)	-	MV repair (not specified)
**Silaschi,** 2024	48/49 (98.0)	1/49 (2.0)	Minimally invasive surgery repair

M-TEER: mitral valve transcatheter edge-to-edge repair; MV: mitral valve.

Supplementary Table 3. Assessment of risk of bias using Cochrane Risk of Bias tool for randomized controlled trials

	EVEREST II Trial, 2011	MATTERHORN Trial, 2024
Random sequence generation (selection bias)	Low risk	Low risk
Allocation concealment (selection bias)	Low risk	Low risk
Blinding of participants and personnel (performance bias)	High risk	High risk
Blinding of outcome assessment (detection bias)	Low risk	Low risk
Incomplete outcome data (attrition bias)	Low risk	Low risk
Selective reporting (performance bias)	Low risk	Low risk
Other bias	Low risk	Low risk

Supplementary Table 4. Assessment of risk of bias using Cochrane Risk of Bias tool In Non-randomized Studies - Interventions, Version 2

	Koschutnik, 2022	Amabile, 2023	Silaschi, 2024
Domain 1: Bias due to confounding	Low	Low	Low
Domain 2: Bias due to selection of participants	Low	Low	Low
Domain 3: Bias in classification of interventions	Low	Low	Low
Domain 4: Bias due to deviations from intended interventions	Low	Low	Low
Domain 5: Bias due to missing data	Moderate	Low	Low
Domain 6: Bias in measurement of outcomes	Low	Low	Low
Domain 7: Bias in selection of the reported result	Moderate	Low	Moderate

Supplementary Table 5. Study population comorbidities

	Hypertension (%)		Diabetes (%)		Coronary Artery Disease (%)		Previous Myocardial Infarction (%)		History of AF or FLA (%)		Previous stroke (%)		History of COPD (%)		GFR, ml/min/1.73 m² (n)	
	M-TEER	Surgery	M-TEER	Surgery	M-TEER	Surgery	M-TEER	Surgery	M-TEER	Surgery	M-TEER	Surgery	M-TEER	Surgery	M-TEER	Surgery
**EVEREST II,** 2011	-	-	14/184 (7.6)	10/95 (10.5)	86/183 (47.0)	44/95 (46.4)	40/183 (21.9)	20/94 (21.3)	59/175 (33.7)	35/89 (39.3)	-	-	27/183 (14.8)	14/95 (14.7)	-	-
**MATTERHORN,** 2024	85/103 (82.5)	86/103 (83.5)	28/103 (27.2)	25/103 (24.3)	48/103 (46.6)	42/103 (40.8)	-	-	57/103 (55.3)	48/103 (46.6)	6/103 (5.8)	7/103 (6.8)	21/103 (35.9)	14/103 (13.6)	57.4±20.8 (99)	56.3±21.5 (98)
**Koschutnik,** 2022	50/51 (98.0)	50/51 (98.0)	17/51 (33.3)	6/51 (11.8)	25/51 (49.0)	17/51 (33.3)	12/51 (23.5)	2/51 (3.9)	36/51 (70.6)	33/51 (64.7)	3/51 (5.9)	5/51 (9.8)	14/51 (27.5)	9/51 (17.6)	58.6±27.0 (51)	68.6±28.2 (51)
**Amabile,** 2023	455/550 (82.7)	453/550 (82.3)	-	-	-	-	-	-	324/550 (58.9)	323/550 (58.7)	190/550 (34.6)*	195/550 (35.5)*	-	-	-	-
**Silaschi,** 2024	-	-	-	-	7/49 (14.3)	4/49 (8.2)	-	-	34/49 (69.4)	20/49 (40.8)	4/49 (8.2)	2/49 (4.1)	12/49 (24.5)	6/49 (12.2)	60.6±27.5 (49)	76.3±29.9 (49)
**Pooled Total**	590/704 (83.8)	589/704 (83.7)	59/338 (17.5)	41/249 (16.5)	166/386 (43.0)	107/298 (35.9)	52/234 (22.2)	22/145 (15.2)	510/928 (55.0)	459/842 (54.5)	203/753 (27.0)	209/753 (27.8)	74/386 (19.2)	43/298 (14.4)	58.5±24.2 (199)	64.4±26.8 (198)

Values are expressed as mean ± standard deviation.
COPD: chronic obstructive pulmonary disease; FA: atrial fibrillation; FLA: atrial flutter; GFR: estimated glomerular filtration rate; M-TEER: mitral valve transcatheter edge-to-edge repair.
* Values displayed regards cerebral artery disease.

Supplementary Table 6. Echocardiographic characteristics of study population

	MR grade 1 (%)		MR grade 2 (%)		MR grade 3 (%)		MR grade 4 (%)		LVEDD, mm (n)		Tricuspid regurgitation ≥ moderate (n)	
	M-TEER	Surgery	M-TEER	Surgery	M-TEER	Surgery	M-TEER	Surgery	M-TEER	Surgery	M-TEER	Surgery
**EVEREST II,** 2011	0/184 (0.0)	0/95 (0.0)	8/184 (4.3)	6/95 (6.3)	130/184 (70.7)	67/95 (70.5)	46/184 (25.0)	21/95 (22.1)	55.3±6.4 (148)	54.1±7.0 (67)	-	-
**MATTERHORN,** 2024	0/102 (0.0)	0/97 (0.0)	3/102 (2.9)	5/97 (5.2)	61/102 (59.8)	54/97 (55.7)	38/102 (37.2)	38/97 (39.2)	61.8±9.5 (95)	60.2±8.7 (85)	-	-
**Koschutnik,** 2022	-	-	-	-	-	-	-	-	52.0±11.5 (51)	51.7±7.8 (51)	28/51 (54.9)	23/51 (45.1)
**Amabile,** 2023	-	-	-	-	-	-	-	-	-	-	-	-
**Silaschi,** 2024	0/24 (0.0)	0/23 (0.0)	2/24 (8.3)	4/23 (17.4)	3/24 (12.5)	4/23 (17.4)	19/24 (79.2)	15/23 (65.2)	51.0±8.0 (49)	50.4±9.4 (49)	29/49 (59.2)	11/49 (22.4)
**Pooled Total**	0/310 (0.0)	0/215 (0.0)	13/310 (4.2)	15/215 (7.0)	194/310 (62.6)	125/215 (58.1)	103/310 (33.2)	74/215 (34.4)	56.0±9.3 (343)	55.0±9.1 (252)	57/100 (57.0)	34/100 (34.0)

Values are expressed as mean ± standard deviation.
LVEDD: left ventricle end-diastolic diameter; MR: mitral valve regurgitation; M-TEER: mitral valve transcatheter edge-to-edge repair.

Supplementary Table 7. Study population therapeutic management

	ACEI/ARB/ARNI at discharge (%)		Beta-blocker at discharge (%)		MRA (%)		CRT (%)	
	M-TEER	Surgery	M-TEER	Surgery	M-TEER	Surgery	M-TEER	Surgery
**EVEREST II,** 2011	-	-	-	-	-	-	-	-
**MATTERHORN,** 2024	83/103 (81.4)	55/95 (57.9)	86/102 (84.3)	79/95 (83.2)	32/102 (31.4)	20/95 (21.1)	16/103 (15.5)	11/103 (10.7)
**Koschutnik,** 2022	34/51 (66.7)	37/51 (72.6)	38/51 (74.5)	42/51 (82.4)	30/51 (58.8)	11/51 (21.6)	-	-
**Amabile,** 2023	-	-	-	-	-	-	-	-
**Silaschi,** 2024	-	-	-	-	-	-	-	-
**Pooled Total**	117/154 (76.0)	92/146 (63.0)	124/153 (81.0)	121/146 (82.9)	62/153 (40.5)	31/146 (21.2)	16/103 (15.5)	11/103 (10.7)

ACEI: angiotensin converting enzyme inhibitor; ARB: angiotensin receptor blocker; ARNI: angiotensin receptor neprilysin inhibition; CRT: cardiac resynchronization therapy; MRA: mineralocorticoid receptor antagonist; M-TEER: mitral valve transcatheter edge-to-edge repair.

Supplementary Figure 1. Sensitivity analysis of 12 months all-cause mortality at 12 months

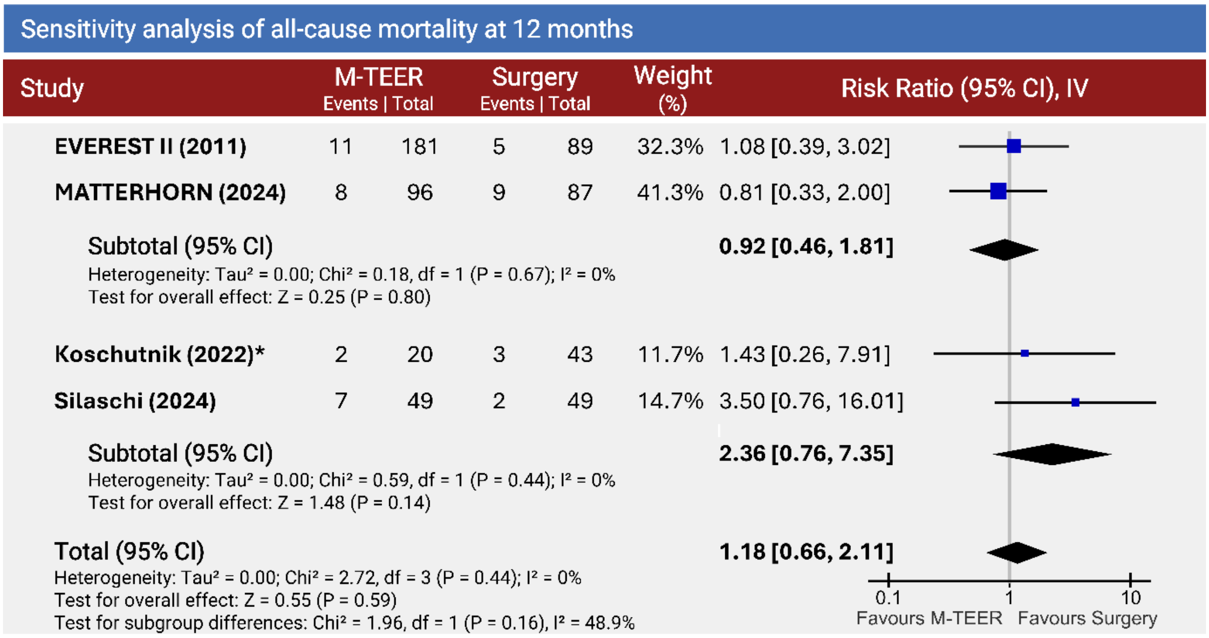

Sensitivity Analysis of All-Cause Mortality at 12 Months. This forest plot presents a sensitivity analysis comparing all-cause mortality between the intervention group mitral transcatheter edge-to-edge repair (M-TEER) and the surgical mitral intervention (valve repair/replacement) at 12 months across multiple studies. Risk ratios (RR) with 95% confidence intervals (CI) are presented for each study and pooled for two subgroups: randomized control trials and observational studies with propensity matched cohorts. The overall pooled analysis demonstrates no statistically significant difference in all-cause mortality between the two groups (RR: 1.18, 95% CI: 0.66–2.11).
*Only includes data from patients with primary mitral regurgitation.

Supplementary Figure 2. Sensitivity analysis of 12 months mitral valve reintervention at 12 months

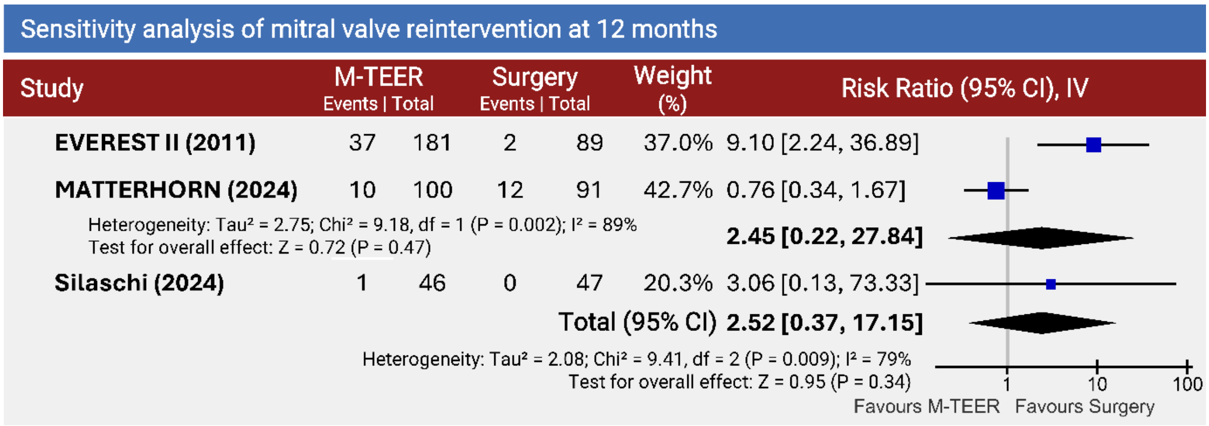

Sensitivity Analysis of Mitral Valve Reintervention at 12 Months. This forest plot presents a sensitivity analysis comparing mitral valve reintervention between mitral transcatheter edge-to-edge repair intervention(M-TEER) and the surgical mitral intervention (valve repair/replacement) at 12 months across multiple studies. Risk ratios (RR) with 95% confidence intervals (CI) are presented for each study and pooled for two subgroups: randomized control trials and observational studies with propensity matched cohorts. The overall pooled analysis demonstrates no statistically significant difference in mitral valve reintervention between the two groups (RR: 2.52, 95% CI: 0.37–17.15).
